# Inhibition of Osteoclast Differentiation and Promotion of Osteogenic Formation by *Wolfiporia extensa* Mycelium

**DOI:** 10.4014/jmb.2304.04048

**Published:** 2023-06-09

**Authors:** Tae Hyun Son, Shin-Hye Kim, Hye-Lim Shin, Dongsoo Kim, Jin-Sung Huh, Rhim Ryoo, Yongseok Choi, Sik-Won Choi

**Affiliations:** 1 School of Life Sciences and Biotechnology, Korea University, Seoul 02841, Republic of Korea; 2 Forest Biomaterials Research Center, National Institute of Forest Science (NIFoS), Jinju 52817, Republic of Korea; 3 Forest Microbiology Division, Department of Forest Bio-Resources, NIFoS, Suwon 16631, Republic of Korea

**Keywords:** Osteoporosis, *Wolfiporia extensa*, mycelium, osteoblasts, osteoclasts

## Abstract

Osteoporosis, Greek for “porous bone,” is a bone disease characterized by a decrease in bone strength, microarchitectural changes in the bone tissues, and an increased risk of fracture. An imbalance of bone resorption and bone formation may lead to chronic metabolic diseases such as osteoporosis. *Wolfiporia extensa*, known as “Bokryung” in Korea, is a fungus belonging to the family *Polyporaceae* and has been used as a therapeutic food against various diseases. Medicinal mushrooms, mycelium and fungi, possess approximately 130 medicinal functions, including antitumor, immunomodulating, antibacterial, hepatoprotective, and antidiabetic effects, and are therefore used to improve human health. In this study, we used osteoclast and osteoblast cell cultures treated with *Wolfiporia extensa* mycelium water extract (WEMWE) and investigated the effect of the fungus on bone homeostasis. Subsequently, we assessed its capacity to modulate both osteoblast and osteoclast differentiation by performing osteogenic and anti-osteoclastogenic activity assays. We observed that WEMWE increased BMP-2-stimulated osteogenesis by inducing Smad-Runx2 signal pathway axis. In addition, we found that WEMWE decreased RANKL-induced osteoclastogenesis by blocking c-Fos/NFATc1 via the inhibition of ERK and JNK phosphorylation. Our results show that WEMWE can prevent and treat bone metabolic diseases, including osteoporosis, by a biphasic activity that sustains bone homeostasis. Therefore, we suggest that *WEMWE* can be used as a preventive and therapeutic drug.

## Introduction

Osteoporosis, a disease that manifests due to impaired bone metabolism, is associated with declining bone density that can lead to fractures, thereby causing severe pain and impairment and reducing the quality of life [1]. Osteoporosis is caused by an imbalance between bone-forming osteoblastogenic and bone-resorbing osteoclastic cells [2]. Therefore, prevention and treatment of osteoporosis is important to lead a better life.

Osteoblasts differentiate from mesenchymal stem cells and play a crucial role in the formation of new bones [3]. These cells are remarkably plastic and the strict regulation of their differentiation is necessary for proper skeletal development and homeostasis [4]. Differentiation of osteoblast is controlled by various intracellular molecules, such as hormones, cytokines, and transcription factors, and this results in bone mineralization and formation [5]. Bone morphogenetic protein 2 (BMP-2) is a primary regulator of osteoblast differentiation that activates downstream signal pathways via Smad 1/5/8 phosphorylation [6]. Osteoblast differentiation is initiated by the development of osteoblasts in osteo-chondroprogenitor cells, which is followed by the activation of osteogenic transcriptional factors, such as runt-related transcription factor 2 (RUNX2), osterix (OSX) and *Drosophila* distal-less 5 (DLX5) [7]. These transcription factors promote the expression of early osteogenic genes, including alkaline phosphatase (ALP) and collagen1α1 chain (COL1A1), which lead to the differentiation of the osteoprogenitors in pre-osteoblasts. It is pertinent to note that the expression of these early osteogenic genes and other osteoblast markers, such as osteopontin (OPN), bone sialoprotein II (BSP II) and osteocalcin (OCN), is sustained throughout the life of mature osteoblasts [8]. As transcription factor Runx2 is an upstream regulator of this differentiation process, it has the highest potential to be a target for novel therapeutic approach to prevent bone loss and promoting bone formation [9].

Osteoclasts, the multinucleated cells that are derived from monocyte/macrophage-lineage cells, are the other important players in bone homeostasis [10] and are involved in bone resorption [11]. Osteoblasts mediate new bone formation through osteoclastogenesis [12], wherein they produce the macrophage colony-stimulating factor (M-CSF), essential for osteoclast differentiation [13]. Another cytokine essential for osteoclastogenesis is receptor activator of nuclear factor-kappa B (NF-κB) ligand (RANKL) [14]. The binding of RANKL to RANK activates a number of signal transduction associated molecules such as NF-κB, Akt, and MAP kinases, which are necessary for the activation of genes essential for osteoclast differentiation [15] such as the c-Fos and the NFATc1 [16-18]. NFATc1, which requires c-Fos for its upregulation, controls osteogenesis by modulating downstream effectors, cathepsin K, dendritic cell-specific transmembrane protein (DC-STAMP) and tartrate-resistant acid phosphatase (TRAP, also known as ACP5) [19, 20]. Consequently, pharmacological inhibition of such transcription factors can potentially be used to overcome diseases associated with impaired bone resorption [21, 22].

Most of the treatments that have been used for decades to prevent and treat bone diseases present side effects [23]. For instance, bisphosphonates, which are used as bone resorption inhibitors [24, 25], can promote necrosis of the jawbone and cause atypical femoral fractures [26]. Therefore, it is crucial to identify and develop new drugs to prevent and treat osteoporosis that have no or minimal side effects. Natural medicines that have been used for centuries in some parts of the world [27] are garnering attention from the scientific community owing to their few/mild side effects [28], which can help in overcoming the aforementioned issues.

Mushrooms comprise two parts: (1) the fruiting body that develops above the ground and (2) the root system, called the mycelium. The mycelium is an organ that absorbs nutrients to facilitate the survival of mushrooms and plays the same role as the root of a plant. Mycelium contains three times the nutrient such a zinc, magnesium and iron than that present in the fruiting body and is essential for fungal growth [29]. Mycelium accumulates higher concentrations of ergosterol and phenolic compounds than the corresponding fruiting body and shows cytotoxicity toward tumor cell lines [30]. Mycelium and fungi are thought to possess approximately 130 medicinal effects, including antitumor, immunomodulating, antioxidant, radical scavenging, cardiovascular, anti-hypercholesterolemic, antiviral, antibacterial, anti-parasitic, antifungal, detoxification, hepatoprotective, and antidiabetic effects [31]. *Wolfiporia extensa*, a fungus in the family *Polyporaceae*, is known as “Bokryung” in Korea. *W. extensa* has been used therapeutically against various diseases [32]. *W. extensa* is a well-known traditional Chinese medicine used for its diuretic, sedative, and tonic effects [33]. Further, it is known to have spleen-invigorative, stomach-tonifying, sedative, tranquilizing, diuretic, and damp-clearing effects [34]. *W. extensa* is mainly used to treat phlegm retention, dysuria, edema, poor appetite, watery stool, palpitations, and insomnia [35]. Additionally, the organic compounds such as U-pachiman, pachymaran, and carboxymethyl pachymaran separated from the Bokryung mycelium have anticancer effects [36, 37], and (1,3)-(1,6)-achymaran has a strong antitumor effect [38]. Furthermore, the triterpene component of Bokryung has anti-inflammatory and anti–skin cancer properties [39], making Bokryung extracts a potentially safe and effective drug candidates.

While its anticancer or anti-inflammatory properties have been studied to a certain extent, the role of *W. extensa* mycelium in bone homeostasis is less explored and therefore we investigated its potential for the treatment of osteoporosis. According to a recent study, ethanol extracts of *W. extensa* can inhibit osteoclastogenesis in vitro and in vivo [40]. However, we observed both osteoclast and osteoblast cells differentiation in vitro. Toward this end, we collected 28 strains of *W. extensa* mycelium comprising 9 cultivated strains, 11 wild strains, and 8 hybrid strains. Given the involvement of *W. extensa* in the regulation of osteoclast differentiation, we screened diverse strain of *W. extensa* that can lead to osteoblast differentiation. We hypothesized that *W. extensa* extracts could play a role in dual action of bone homeostasis. We observed that *W. extensa* can enhance osteoblast differentiations and inhibit osteoclast differentiation, which could help in the development of new therapeutic drugs that can maintain bone homeostasis, thereby opening new avenues in the prevention and treatment of osteoporosis.

## Material and Methods

### Preparation of the *Wolfiporia extensa* Mycelium Extract

We collected 28 strains of *Wolfiporia extensa* mycelia which consisted of 9 cultivated strains, 11 wild strains, and 8 hybrid strains. The mycelia were incubated at 25°C for 60 days with a 2-liter potato dextrose broth medium in a 5-liter triangular flask following which they were extracted using hot water and 100% ethanol. We used an evaporator to remove all the solvents and measured the weight of *W. extensa* mycelium extracts. We reconstituted the *W. extensa* mycelium water extracts (WEMWE) using 30 mg/ml dimethyl sulfoxide (DMSO).

### Reagents and Antibodies

recombinant human bone morphogenetic protein-2 (rhBMP-2), Mouse-soluble receptor activator of nuclear factor-κB ligand (RANKL) and macrophage colony-stimulating factor (M-CSF) were purchased from R&D Systems (USA) and reconstituted in 0.1% bovine serum albumin that was dissolved in Dulbecco’s phosphate buffered saline (DPBS) according to the used dose. DPBS was purchased from Cytiva (USA). Trizol reagent was purchased from Invitrogen/Thermo Scientific (USA) ALP antibodies (AF2910) Antibody was purchased from R & D Systems. Antibodies for NFATc1 (sc-7294), c-Fos (sc-271243), Smad (sc-398844), Actin (sc-47778) and the Anti-mouse and rabbit antibody conjugated to horseradish peroxidase (HRP) were purchased from Santa Cruz Biotechnology (USA). All other antibodies were purchased from CST (USA).

### Ethics Statement for Animal Works

The use of experimental animals was approved, and this study was performed in accordance with the National Institute of Health (NIH) Guide for the Care and Use of Laboratory Animals and the Institutional Animal Care and Use Committee of Yonsei University College of Medicine (Permit no. 2022-0104).

### Preparation of Bone Marrow-Derived Macrophage

All experiments were carried out as described in a previous study [41]. To obtain BMM cells from 5- to 8-week-old male ICR mice (Orient Bio, Korea), we flushed their femur and tibia of the leg with α-MEM supplemented with antibiotics. For one day, the bone marrow cells were spread on 10 cm cell culture dish in α-MEM media contained with 10% FBS and M-CSF (10 ng/ml). The cells that didn't adhere to the culture dish were moved onto the other culture dishes. The cells transferred cultured for 3 days in α-MEM media including M-CSF (30 ng/ml). After non-attached cells were washed away, and cells attached to the culture dish were harvested and used as BMMs.

### Osteoclast Differentiation

For osteoclast differentiation, BMMs were seeded at a density of 10^4^ cells/well in a 96-well plate or 3 × 10^5^ cells/well in a 6-well plate and cultured with M-CSF (50 ng/ml) and RANKL (30 ng/ml) for 4 days. All media changed every 3 days. Multinucleated osteoclasts were then observed by an inverted microscope.

### Tartrate-Resistant Acid Phosphatase Staining and Activity Assay

Differentiated Osteoclasts were confirmed using TRAP staining, a marker of osteoclast differentiation. Mature osteoclasts were fixation to 3.7% formaldehyde for 5 min, rinsed with distilled water, then stained with TRAP Staining Kit (USA). We redefined TRAP-positive cells are 3 nuclei cells at least. Therefore TRAP-positive multinucleated osteoclasts (MNC; nuclei ≥ 3) were counted through an inverted microscope. To measure TRAP activity a marker of mature osteoclast, multinucleated osteoclasts were fixed in 3.7% formaldehyde for 5 min, rinsed with distilled water, and treated with TRAP buffer (100 mM sodium citrate, pH 5.0, 50 mM sodium tartrate) supplemented with 3 mM p-nitrophenyl phosphate (PNPP), substrate of TRAP (Sigma-Aldrich, USA) at room temperature for 20 min. After the reaction, TRAP buffers were moved to the other plates filled with 50 μl of 0.1 N NaOH as stop solution. Optical density was determined at 405 nm by spectrophotometer (Molecular Devices, USA; SpectraMax iD3).

### Cell Cytotoxicity Assay

The Cell Counting Kit-8 (CCK-8) assay was performed to examine the cytotoxic effect of WEMWE on BMMs and C2C12 cells. Cells were seeded in a 96-well plate then cultured for 3 days with various concentrations of WEMWE. After the incubation period, cell cytotoxicity was determined using the CCK-8 (Dojindo Molecular Technologies, USA). Next, the cells were added to media containing CCK-8 solution for 30 min. Optical density was determined at 450 nm using spectrophotometer (Molecular Devices; SpectraMax iD3).

### RNA Extraction and Quantitative Reverse Transcription Polymerase Chain Reaction

Quantitative reverse transcription polymerase chain reaction (qRT-PCR) was run as previously described [41]. Primer pairs were made using the online Primer3 program [42]. Table S1 summarizes the primer sets used in this study. Briefly, the total RNA from BMM and C2C12 cells were extracted from the cultures using TRIzol reagent according to the manufacturer’s protocol. cDNA was prepared from 1 μg total RNA using the RevertAid First Strand cDNA Synthesis Kit (Thermo Scientific) according to the manufacturer’s protocol for quantitative reverse transcription polymerase chain reaction (qRT-PCR). SYBR green-based qRT-PCR was run using the QuantStudio 5 real-time PCR System (Thermo Scientific) and PowerUp SYBR Green Master Mix (Thermo Scientific). All sample mixtures were run in three replicates, and the data were analyzed using the 2^–ΔΔCT^ method as described in Livak and Schmittgen [43]. Glyceraldehyde 3-phosphate dehydrogenase (*Gapdh*) was used as internal control.

### Western Blot

Immunoblot analysis was performed as previously described [41]. After WEMWE or vehicle treated cells were lysed in Ripa lysis buffer (Cell signaling technology) containing protease inhibitors. After 10 min in ice, centrifuge at 15,000 ×*g* for 15 min, the proteins were harvested at supernatant. The concentration of protein lysates was measured detergent compatible (DC) protein assay kit (Bio-Rad, USA). The proteins were subjected to a SDS-PAGE gel electrophoresis and transferred to PVDF membranes (Merck Millipore, Germany). Then membranes were incubated with the relevant primary and HRP-coupled secondary antibodies and developed using Clarity Western ECL Substrate (Bio-Rad) and visualized with the ChemiDoc XRS+ (Bio-Rad). Gapdh and Actin were used as internal controls.

### Osteoblast Differentiation

All osteoblast-associated experiments were performed as previously described with modifications [41]. C2C12 cells were plated on 96-well plates at 2.5 × 10^3^ cells/well or in 6-well plates at 2.5 × 10^5^ cells/well and maintained in α-MEM containing 10% FBS and antibiotics. After one day in culture, cells media were changed to the α-MEM supplemented with 5% FBS and recombinant human BMP-2 (rhBMP-2; 50 ng/ml) for osteoblast differentiation.

### Alkaline Phosphatase Staining and Activity Assays

osteoblast differentiation is expressed ALP in early time; hence we used ALP staining to assess osteoblastic bone formation. Toward this end, C2C12 cells differentiated for 3 days in vitro were removed media, and fixation with 3.7% formaldehyde for 5 min, rinsed with distilled water, and added up an BCIP/NBT Liquid Substrate System (Sigma-Aldrich). To determine ALP activity, after BMP-2 treated cells were fixation with 3.7% formaldehyde for 5 min, rinsed with distilled water, and then treated with 1-Step PNPP Substrate Solution (Thermo Scientific) at room temperature for 20 min. The PNPP Solution that's done with the reaction were moved to the other plates filling 50 μl of 0.1 N NaOH as stop solution, and the optical density was measured at 405 nm using a spectrophotometer (Molecular Devices; SpectraMax iD3).

### Statistical Analysis

**Fig. 1:**
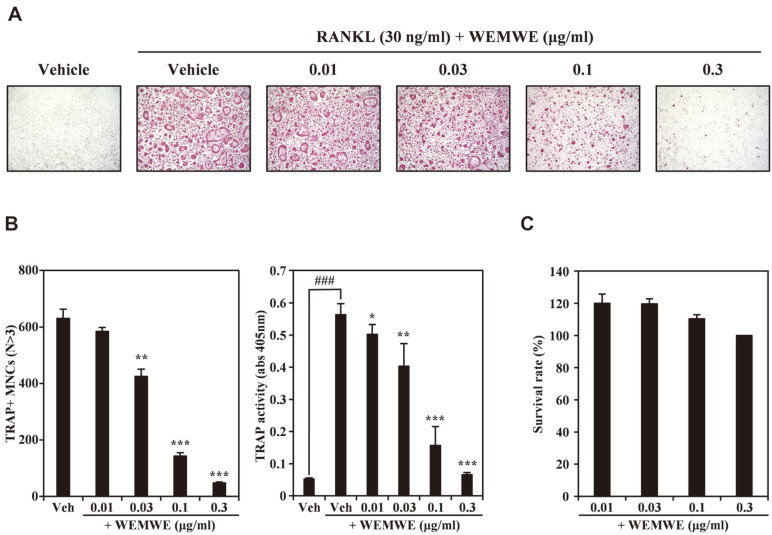
*Wolfiporia extensa* mycelium water extract (WEMWE) impairs RANKL-mediated osteoclast differentiation. (**A**) After overnight cell seeding, BMM cells were treated for 4 days with M-CSF (50 ng/ml) and RANKL (30 ng/ml) in the presence of WEMWE (0.01, 0.03, 0.1, and 0.3 μg/ml) or vehicle (dimethyl sulfoxide; DMSO). Multinucleated osteoclasts were visualized using TRAP staining. (**B**) TRAP-positive multinuclear cells were counted using an inverted microscope (left panel) and TRAP activity was measured using a spectrophotometer (right panel). *p < 0.05; **p < 0.01; ***p < 0.001 (versus vehicle control). (**C**) Effect of WEMWE on the viability of BMMs was evaluated using the CCK-8 assay.

**Fig. 2:**
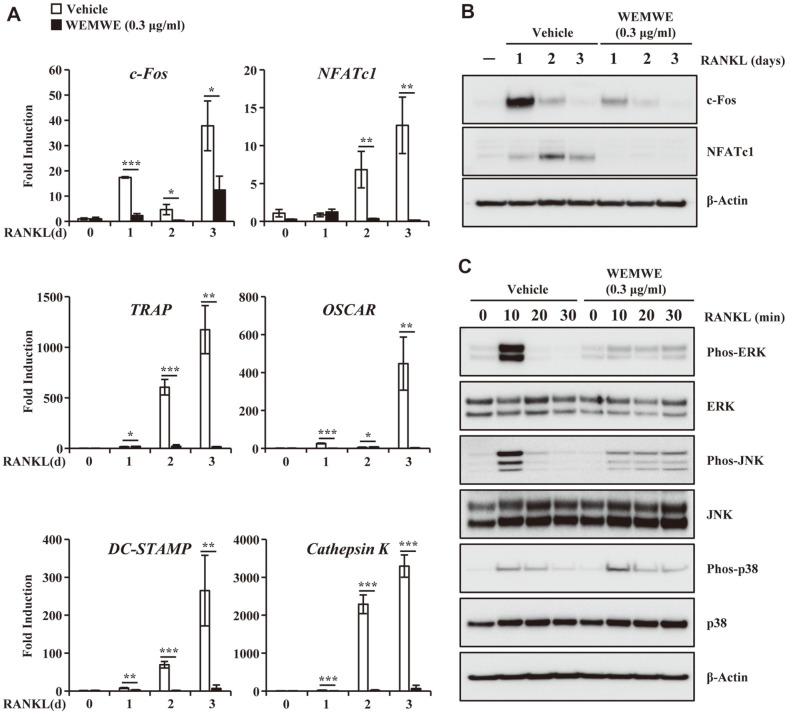
WEMWE inhibits RANKL-induced expression of c-Fos/NFATc1 by modulating ERK and JNK phosphorylation. (**A**) Total RNA of BMM cells that had finished differentiation was isolated using TRIzol reagent and mRNA expression was measured using real-time PCR. *Gapdh* was used as an internal control. (**B**) The effect of WEMWE on the protein expression level of RANKL-induced transcription factors was evaluated using Western blot analysis. Actin was used as an internal control. (**C**) The indicated signaling molecules expression levels were quantified using Western blot analysis. Following serum starvation for 1 d, BMM cells were pre-treated with vehicle or WEMWE (0.3 μg/ml) for 1 h prior to RANKL stimulation (30 ng/ml) for the indicated times. Actin was used as an internal control. One representative result from three independent experiments yielding similar results is shown. The experiment was performed in triplicate. *p < 0.05; **p < 0.01; ***p < 0.001 (versus vehicle control).

**Fig. 3:**
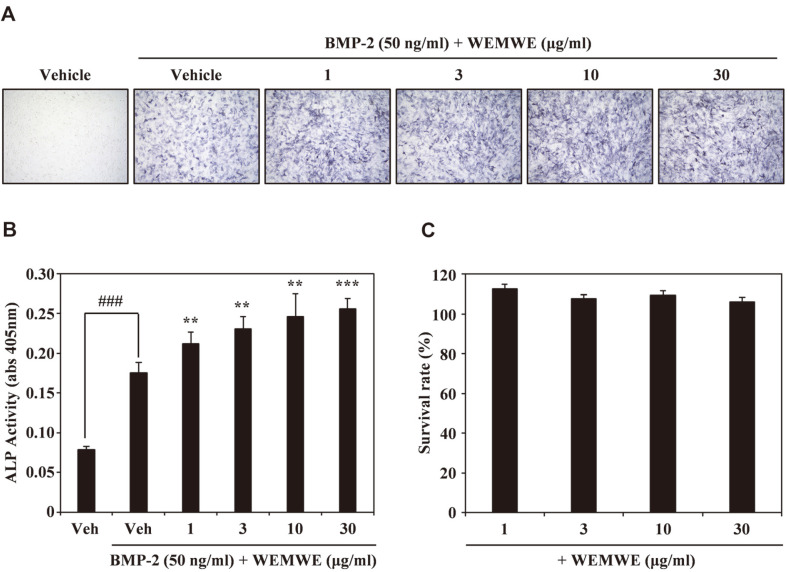
WEMWE promotes BMP-2-induced osteoblast differentiation. (**A**) After overnight cell seeding, C2C12 cells were treated for 4 days with vehicle (DMSO) or WEMWE (1, 3, 10, and 30 μg/ml) in the presence of BMP-2 (50 ng/ml). Osteoblast differentiation was visualized via alkaline phosphatase staining. (**B**) ALP activity was monitored by measuring absorbance at 405 nm. (**C**) Effect of WEMWE on the viability of C2C12 cells was evaluated using the CCK-8 assay.

**Fig. 4:**
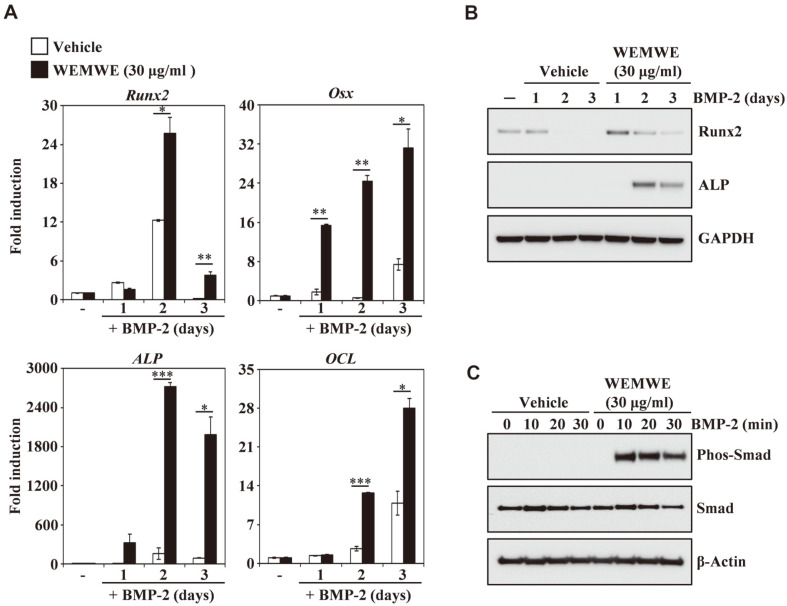
WEMWE stimulates BMP-2–induced expression of Runx2. (**A**) C2C12 cells were stimulated in the presence of BMP-2 (50 ng/ml) with either vehicle (water) as a control or WEMWE (30 μg/ml) for the indicated times. The mRNA expression levels were assessed using real-time PCR. GAPDH was used as an internal control. The sample was performed triplicate. *p < 0.05; **p < 0.01; ***p < 0.001 (versus vehicle control). (**B**) Effects of WEMWE on the expression levels of Runx2 and ALP were evaluated by immunoblot analysis. GAPDH was used as an internal control. (**C**) WEMWE induces BMP-2– mediated phosphorylation of Smad signaling molecules. Following 24 h serum starvation, C2C12 cells were pre-treated with vehicle as a control or WEMWE (30 μg/ml) for 1 h prior to BMP-2 stimulation (50 ng/ml) for the indicated times. The expression levels of the signaling molecules were evaluated by Western blotting. Actin was used as an internal control. One representative result from three independent experiments yielding similar results is shown.

All quantitative values are presented as mean ± standard deviation. Each experiment included three replicates for each experimental variable and was performed three to five times. Figs. 1–4 show the results from one representative experiment. Statistical differences were analyzed using the Student’s t-test, and a value of *p* < 0.05 was considered significant.

## Results

### WEMWE Inhibits RANLK-Induced Osteoclast Differentiation in BMM Cells

We screened 28 strains of *Wolfiporia extensa* mycelia, which consisted of 9 cultivated strains, 11 wild strains, and 8 hybrid strains, for dual action of bone homeostasis. We found that one of the water extract fractions of the mycelium had the most potential (data not shown). We further investigated the mode-of-action of this WEMWE on osteoclastogenesis and osteoblast differentiation. First, to examine the effect of WEMWE on RANKL-induced osteoclast differentiation, osteoclasts differentiated from BMMs through RANKL treatment were incubated with increasing concentrations of WEMWE. After three days of WEMWE application, mature osteoclasts were assessed in each group through TRAP staining. Initially, we observed that the TRAP staining decreased with increased concentration of WEMWE, suggesting that WEMWE inhibits development of TRAP-positive multinuclear bone cells (TRAP^+^ MNCs) in dose reliance (Fig. 1A). This decrease pattern was known by quantification of the number of TRAP^+^ MNCs (from 630 cells to 47 cells, P value of WENWE treatment compared with control is lower than 0.001 score; Fig. 1B, left panel) and by confirming TRAP activity (Fig. 1B; right panel). Notably, highest concentration of WEMWE (0.3 μg/ml) showed a strong inhibitory effect as only 10% of the cells remained TRAP^+^ compared to control cells which were treated with vehicle alone. To eliminate the potential cytotoxic effects of WEMWE on osteoclast formation, cell viability of BMMs were evaluated simultaneously using CCK-8 analysis. We observed that WEMWE treated cells did not exhibit any sign of cytotoxicity at the WEMWE doses used in this study (0.01 μg/ml: 120%, 0.03 μg/ml: 119%, 0.1 μg/ml: 110%, 0.3 μg/ml: 100%; Fig. 1C). These results suggest that WEMWE significantly attenuate RANKL-induced osteoclast differentiation with no apparent cytotoxicity.

### WEMWE Inhibits Osteoclast Differentiation by Modulating MAPK and AP-1 Transcription Factor

To further delineate the mechanism through which WEMWE inhibits osteoclast differentiation we probed for the expression of genes, including transcriptional factors, necessary for the differentiation program through qRT-PCR. We observed that WEMWE 0.3 μg/ml inhibited RANKL-induced mRNA levels of *c-Fos* and *NFATc1*, and osteoclastogenesis marker genes such as TRAP, Osteoclast-associated receptor (OSCAR), Dendritic cell-specific transmembrane protein (DC-STAMP), and cathepsin K (Fig. 2A). Additionally, immunoblot analysis confirmed that RANKL-induced protein levels of c-Fos and NFATc1 was further attenuated by WEMWE at dose 0.3 μg/ml (Fig. 2B). To decipher the signaling pathways impacted by WEMWE treatment, we performed Western blot analysis of osteoclasts differentiated through RANK which were treated with increasing concentration of WEMWE and compared the ratio of phosphorylation of several proteins to their total proteins to untreated cells. Interestingly, cells treated with WEMWE exhibited a slight decrease in the expression levels of Phos-ERK and Phos-JNK compared to control cells while Phos-P38 did not show a significant change in its expression (Fig. 2C). These results indicate that the inhibition of ERK and JNK phosphorylation could contribute to anti-osteoclast differentiation activity.

### WEMWE Enhances BMP-2-Induced Osteogenesis in C2C12 Cells

Osteoblasts play a major role in bone production [44]. BMP-2 promotes the differentiation of osteoblasts by inducing ALP expression and activity in C2C12 cells [45]. Therefore, we used ALP staining and activity to probe the osteoblastic effect of WEMWE on BMP-2-induced osteoblast differentiation. WEMWE treatment compared with control showed more stained cells, which has blue-purple color. Our results indicate that WEMWE dose-dependently increased BMP-2-mediated ALP expression (Fig. 3A). Furthermore, WEMWE and BMP-2 treatment group was 47% higher than only BMP-2 treat group. WEMWE remarkably increased ALP activity by BMP-2 in dose reliance (Fig. 3B). We further confirmed that through the quantification of the number of surviving cells that the increasing doses of WEMWE used in this study is not accompanied by cytotoxicity (Fig. 3C). Taken together, our results suggest that WEMWE enhances the differentiation of osteoblasts into C2C12 cells.

### WEMWE Enhances the Expression of Osteoblast Differentiation Markers Runx2 and its Downstream Partners

We next investigated mechanism of WEMWE on BMP-2-induced mRNA expression of Runx2/Osx and BMP-2 signaling pathway target gene involved in osteoblast differentiation. We observed that Runx2 was synergistically enhanced when treated with WEMWE (Fig. 4A, top left panel). Furthermore, the expression of Runx2/Osx downstream partners, ALP and OCL, were also elevated in their expression upon WEMWE treatment compared to control. We also performed Western blotting to assess whether the increased mRNA expression observed upon WEMWE treatment led to increase in protein expression. We found that BMP-2 induced Runx2 protein expression was enhanced by the addition of WEMWE following 24 h of treatment, and lasted longer than in control cells. In addition, ALP expression was detectable after 48 h of WEMWE treatment, indicating that WEMWE could regulate the Runx2-ALP pathway (Fig. 4B). These results indicate that WEMWE has latent ability to activate the protein and mRNA level of Runx2 necessary for osteoblast differentiation. Osteoblast differentiation induced BMP-2 through Runx2 and Smad interactions, which contributed to the in vivo bone formation [46]. We further observed that WEMWE induced BMP-2-stimulated phosphorylation of Smad (Fig. 4C). Therefore, our results suggest that WEMWE increased BMP-2-dependent osteoblasts differentiation by enhancing Smad-Runx2 signaling and thus potentially affecting bone formation.

## Discussion

In this study, we aimed to delineate the potential role of WEMWE in bone homeostasis. Subsequently, our study identifies *W. extensa* as a potential therapeutic agent to prevent and treat bone diseases, as we show that WEMWE inhibits RANKL-induced osteoclast differentiation by attenuating TRAP activity and increases BMP-2-dependent osteoblast differentiation by inducing ALP expression with no apparent cytotoxicity.

Mature osteoclast cells play a crucial role in bone resorption [11]. Numerous factors such as cytokines, signaling molecules and transcription factors are involved in osteoclast differentiation [47]. M-CSF, RANKL are produced by osteoblasts and play a vital role in the activation of osteoclast differentiation [47]. In the present study, we found that WEMWE significantly attenuated RANKL-induced osteoclastogenesis without an accompanied cytotoxicity, which can inhibit bone reabsorption by inhibiting osteoclast differentiation. We next examined the molecular markers involved in osteoclast differentiation to establish the mechanism with which WEMWE inhibits osteoclast differentiation. In BMMs, stimulation of RANKL induces the expression major transcription factors involved in osteoclast differentiation, notably, NFATc1, an important regulator of osteoclast differentiation is controlled by c-Fos, which is showed in initial phase of osteoclast differentiation [48, 49]. We observed that WEMWE treatment reduced RANKL-stimulated expression of c-Fos and NFATc1, and downstream genes TRAP, OSCAR, DC-STAMP, and cathepsin K. For osteoclastogenesis, RANKL accelerates different signal transductions containing NF-κB, PI3K/AKT, and MAP kinases such as ERKs, JNKs, and p38 [50]. MAP kinases are important signaling molecules that can change c-Fos and NFATc1 transcription factor in RANKL signaling [50]. In this study, we observed an inhibitory effect of WEMWE on osteoclastogenesis by potentially attenuating the ERK/JNK-c-fos/NFATc1 signaling cascade during osteoclast differentiation. WEMWE decreases RANLK-induced osteoclast differentiation in BMM cells by modulating MAPK and AP-1 transcription factor. The attenuated transcription factor associated with osteoclast differentiation by WEMWE leads to reduction of levels of downstream effectors OSCAR, TRAP, cathepsin K, and DC-STAMP.

Previous work demonstrated that BMP-2 promotes the differentiation of multipotent mesenchymal precursor cells, C2C12, into preosteoblasts which have the capability for bone formation and mineralization [51]. Additionally, BMP-2 induced differentiation of C2C12 cells into osteoblasts leads to ALP expression, an early marker of osteoblastic differentiation [51]. In our study, we observed that WEMWE dramatically increased the expression of ALP at both mRNA and protein levels during BMP-2-induced osteoblast differentiation.

To better understand the mechanism associated with enhancement of osteoblast differentiation by WEMWE, we demonstrated mechanisms involved in BMP-2-induced osteogenesis. Runx2/Osx, a transcriptional activator downstream of BMP signaling, regulates the expression of molecules associated osteoblast differentiation, including ALP and OCL, and is essential for osteogenesis, bone synthesis, and maintenance [52]. We observed that Runx2/Osx, and its downstream effectors, ALP and OCL, were upregulated in cells treated with WEMWE. To understand the mechanisms through which WEMWE enhanced osteoblast differentiation when Runx2 is highly expressed, we evaluated whether WEMWE affected the activation of BMP-2-mediated signaling markers. Smad proteins play a central role in relaying the BMP signal from the receptor to target genes in the nucleus [53] and are essential for bone formation. The important step in BMP-2-signal pathway is the activation of Smad1, Smad5, and Smad8 (Smad 1/5/8) [54]. Interestingly, WEMWE treatment enhanced the activation of Smad, a major BMP2 regulated molecular marker that is required for osteoblast differentiation. WEMWE increases BMP-2-induced osteoblast differentiation in C2C12 cells through the induction of the Smad-Runx2/Osx signaling axis. Thus, the upregulation of Runx2 and Osx could potentially enhance the bone anabolic activity through the downstream effectors ALP, and OCL as well as phosphorylation of Smad.

In conclusion, our study demonstrates that *W. extensa* mycelium extract has dual effect to bone homeostasis: decrease of osteoclast formation and activation of osteoblast differentiation. Taken together, our results indicate that the obvious anti-osteoporotic effect of WEMWE exhibits promising therapeutic potential for metabolic diseases of the bone including osteoporosis. However, animal experiments did not confirm these results; therefore, further animal studies are warranted. Furthermore, further experiments are needed to identify the exact molecules in WEMWE that produce anti-osteoporosis activity and could be used as pharmaceutical compounds. Overall, based on the findings, we suggest that WEMWE can be used as a preventive and therapeutic drug for bone homeostasis disease such an osteoporosis.

## Supplemental Materials

Supplementary data for this paper are available on-line only at http://jmb.or.kr.
