# Supplementary material for: Inhibition of Osteoclast Differentiation and Promotion of Osteogenic Formation by Wolfiporia extensa Mycelium
Source: J Microbiol Biotechnol. 2023 Jun 9;33(9):1197–205. doi: 10.4014/jmb.2304.04048 (PMC10580891; doi:10.4014/jmb.2304.04048)
Supplement: Supplementary file 1 [file jmb-33-9-1197-supple.pdf]

J. Microbiol. Biotechnol.
https://doi.org/10.4014/jmb.2304.04048

Supplementary Table 1.

Target Gene	Forward Primer (5′–3′)	Reverse Primer (5′–3′)
c-Fos	CCAGTCAAGAGCATCAGCAA	AAGTAGTGCAGCCCGGAGTA
NFATc1	GGGTCAGTGTGACCGAAGAT	GGAAGTCAGAAGTGGGTGGA
TRAP	GATGACTTTGCCAGTCAGCA	ACATAGCCCACACCGTTCTC
OSCAR	AGGGAAACCTCATCCGTTTG	GAGCCGGAAATAAGGCACAG
DC-STAMP	CCAAGGAGTCGTCCATGATT	GGCTGCTTTGATCGTTTCTC
Cathepsin K	GGCCAACTCAAGAAGAAAAC	GTGCTTGCTTCCCTTCTGG
Runx2	GACTGTGGTTACCGTCATGGC	ACTTGGTTTTTCATAACAGCGGA
ALP	GATGGCGTATGCCTCCTGCA	CGGTGGTGGGCCACAAAAGG
OCL	AGGAAACCTCATCCGRRG	GAGCCGGAAATAAGGCACAG
Osx	CTTCCACTTCGCCTGCACCC	GGAGCATAGGAACTAGGCAC
HPRT1	TGCTCGAGATGTCATGAAGG	AGAGGTCCTTTTCACCAGCA
GAPDH	ACCACAGTCCATGCCATCAC	TCCACCACCCTGTTGCTGTA
